# Synergistic Anti-Tumor Activity by Targeting Multiple Signaling Pathways in Ovarian Cancer

**DOI:** 10.3390/cancers12092586

**Published:** 2020-09-10

**Authors:** Wei Wen, Ernest S. Han, Thanh H. Dellinger, Leander X. Lu, Jun Wu, Richard Jove, John H. Yim

**Affiliations:** 1 Department of Surgery, City of Hope National Med Center, Duarte, CA 91010, USA; ehan@coh.org (E.S.H.); tdellinger@coh.org (T.H.D.); laker24lxl@gmail.com (L.X.L.); 2 Department of Molecular Medicine, City of Hope National Med Center, Duarte, CA 91010, USA; Jove.richard@gmail.com; 3 Department of Comparative Medicine, City of Hope National Med Center, Duarte, CA 91010, USA; jwu@coh.org

**Keywords:** sunitinib, dasatinib, everolimus, targeted therapy, combination, ovarian cancer

## Abstract

**Simple Summary:**

Ovarian cancer remains the most lethal gynecological cancer in women. There is a critical need to develop novel strategies that can be used to improve the survival of patients with advanced and recurrent ovarian cancer. Preclinical and early clinical studies with single-targeted agents have shown limited antitumor activity in ovarian cancer. In this study, we found that combined treatment of several FDA-approved targeted drugs—sunitinib, dasatinib, and everolimus—results in simultaneous inhibition of multiple signaling pathways and a better anti-tumor activity than any single treatment. This combination also significantly improves efficacy of paclitaxel in human ovarian cancer. This study may provide a potential combination therapy for the treatment of advanced ovarian cancer.

**Abstract:**

More effective therapy is needed to improve the survival of patients with advanced and recurrent ovarian cancer. Preclinical and early clinical studies with single molecular targeted agents have shown limited antitumor activity in ovarian cancer, likely due to compensation by alternative growth/survival pathways. An emerging strategy in overcoming resistance is to combine inhibitors targeting multiple pathways. In this study, we used a novel strategy of combining several FDA-approved targeted drugs, including sunitinib, dasatinib, and everolimus, in human ovarian cancers. Combination of the tyrosine kinase inhibitor sunitinib with the SRC inhibitor dasatinib showed synergistic anti-tumor activity in human ovarian cancer cells. The increased activity was associated with inhibition of the STAT3, SRC, and MAPK signaling pathways, but not AKT signaling. To inhibit the PI3K/AKT/mTOR pathway, we added the mTOR inhibitor everolimus, which further increased anti-tumor activity in cells. Combined treatment with sunitinib, dasatinib, and everolimus also resulted in greater inhibition of human ovarian tumor growth in mice. Furthermore, the triple combination also synergistically increased the anti-tumor activity of paclitaxel, both in vitro and in vivo. Taken together, our results demonstrate that simultaneous inhibition of several signaling pathways results in better anti-tumor activity compared to inhibiting any of these signaling pathways alone.

## 1. Introduction

Ovarian cancer remains the most lethal gynecological cancer in women. It is typically diagnosed at an advanced stage when the cancer has already spread into the peritoneal cavity [1,2,3]. The current first-line treatment for ovarian cancer is cytoreductive surgery followed by taxane-platinum chemotherapy [3,4,5,6]. Maximum cytoreductive surgery is associated with the longest patient survival, especially when combined with intraperitoneal chemotherapy [7]. Although the initial response rate to standard therapy is greater than 70%, the majority of patients will eventually relapse and require further treatment [5]. Clinical studies of several chemotherapy agents, either alone or in combination, have demonstrated moderate response in patients with advanced and recurrent ovarian cancer. Increased understanding of the molecular events underlying ovarian cancer has led to the development of several molecular targeted therapies in ovarian cancer [8,9]. The most promising therapies in the clinic at this time are those directed toward inhibition of angiogenesis or of DNA repair (e.g., PARP inhibitor). Both types of inhibitors show encouraging progression-free survival (PFS) benefits [10]. Other potential therapeutic targets include cell growth/survival pathways and immune checkpoints [11].

Increasing evidence suggests that ovarian cancer is a highly heterogeneous disease with complex molecular and genetic changes [12]. Analysis of the Cancer Genome Atlas data indicates that mutation in oncogenic driver genes is rare in ovarian cancer. There is no predominant pathway that is deregulated in most ovarian cancer patients. Instead, the concurrent activation of multiple signaling pathways, including PI3K/AKT/mTOR, SRC, MEK/MAPK, and JAK/STAT3, appears to be more common in ovarian cancer and may play an important role in ovarian tumor growth [9,13,14,15]. Various small molecule inhibitors targeting these pathways, including sunitinib, dasatinib, and everolimus, have been developed. However, clinical trials with single-targeted agents have shown limited antitumor activity in ovarian cancer, which could be due to compensation by alternative growth/survival pathways. Thus, an emerging strategy for overcoming resistance is to combine inhibitors targeting multiple oncogenic pathways [16].

Sunitinib (Sutent) is a United States Food and Drug Administration (FDA)-approved agent for the treatment of renal cell carcinoma (RCC), pancreatic neuroendocrine tumors (PNET), and imatinib-resistant gastrointestinal tumors (GIST) [17,18]. Sunitinib blocks several receptor tyrosine kinases relevant to tumor angiogenesis, including vascular endothelial growth factor (VEGF) receptors and platelet-derived growth factor (PDGF) receptors [19]. Sunitinib also inhibits receptors such as c-KIT, FLT3, and RET, which are important for the growth of solid tumors and hematologic malignancies [20]. As a multi-targeted tyrosine kinase inhibitor, sunitinib also inhibits STAT3 in tumor cells such as renal and medulloblastoma tumor cells [21,22]. Phase I and II studies of sunitinib in epithelial ovarian cancer have shown that it demonstrates acceptable toxicity but modest activity [23].

Dasatinib is a SRC kinase inhibitor approved by the FDA for the treatment of imatinib-resistant or -intolerant adult chronic myeloid leukemia and Philadelphia chromosome-positive acute myeloid or lymphoblastic leukemia [24]. The SRC family of kinases is a family of non-receptor tyrosine kinases that regulate several signaling pathways that impact on the behavior of tumor cells, including proliferation, survival, invasion, and angiogenesis [25]. Increased SRC activity has been found in ovarian cancer cell lines and late-stage, poor-prognosis ovarian tumors [26,27]. Preclinical studies have demonstrated the anti-tumor activity of SRC inhibitors in several cancers, including prostate, colon, breast, and ovarian [28,29,30], but their efficacy in clinical trials has not been adequate as single agents for recurrent high-grade serous ovarian cancer (HGSOC), with only 21% of patients being progression-free after 6 months [31,32].

Everolimus is a mammalian target of rapamycin (mTOR) inhibitor approved by the FDA for use in advanced RCC, PNET, and breast cancer [33,34,35]. The PI3K/AKT/mTOR pathway is a signal transduction pathway that links response to growth-related hormone receptor interaction to downstream targets, such as AKT and mTOR [36,37,38]. This pathway affects cell proliferation, survival, and apoptosis [39]. Combination treatment targeting the PI3K/AKT/mTOR pathway with mTOR inhibitors in patients with alterations in the PI3K/AKT/mTOR pathway results in significantly better outcomes in both treatment-naïve and previously treated patients [40]. However, when used alone, mTOR inhibitors have limited efficacy [41,42]. Investigations of mTOR inhibitors in combination with a variety of therapies are underway in ovarian cancer. For example, a 47% progression-free survival rate was reported among 19 evaluable patients treated with everolimus and letrozole in a phase II trial [43]. Trials that included ovarian cancer patients among their inclusion criteria and incorporated everolimus into a dual-treatment regimen showed tolerability with some responses.

In this study, we investigated a novel strategy involving combination of several FDA-approved targeted drugs, including sunitinib, dasatinib, and everolimus, in human ovarian cancers. Our results demonstrate that combined treatment leads to concurrent inhibition of multiple signaling pathways and a greater inhibition of tumor growth, both in vitro and in vivo.

## 2. Results

### 2.1. Combined Treatment with Sunitinib and Dasatinib Leads to Inhibition of Multiple Signaling Pathways

**Figure 1:**
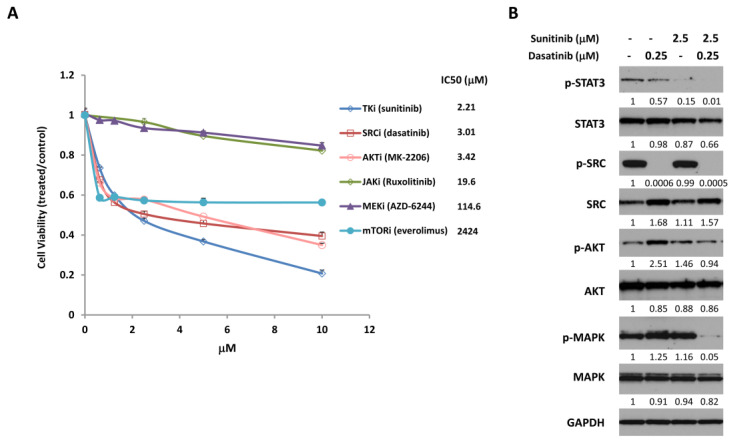
Sunitinib and dasatinib inhibit cell proliferation and cell signaling. (**A**) SKOV3 human ovarian cancer cells were treated with the indicated concentrations of various targeted inhibitors. Cell viability was determined 72 h later. IC50s were determined using the Graphpad Prism. (**B**) SKOV3 cells were treated with the indicated concentrations of sunitinib, dasatinib, or the combination for 24 h and analyzed by Western blot for the expression of phosphorylated and total forms of STAT3, SRC, AKT and MAPK. GAPDH was used as a loading control. Numbers below the corresponding blot represent densitometric analysis normalized to GAPDH. Results are representative of at least three preparations.

To investigate the efficacy of combining inhibitors that target various growth signaling pathways in ovarian cancer, we first tested their individual effects on the proliferation and viability of SKOV3 ovarian cancer cells. We incubated cells with increasing concentrations of a multi-targeted tyrosine kinase inhibitor (sunitinib), an SRC inhibitor (dasatinib), an AKT inhibitor (MK-2206), a JAK inhibitor (ruxolitinib), a MEK inhibitor (AZD-6244), or an mTOR inhibitor (everolimus). We measured cell viability 72 h later, which showed that all of the inhibitors reduced cell viability to some degree, with IC50s (i.e., concentration of drug that gives 50% inhibition) ranging from 2.21 µM to 2424 µM (Figure 1A).

The relatively modest effect of each of these inhibitors on cell survival could be due to the activation and compensation of multiple survival pathways. Several survival pathways are persistently activated in ovarian cancer cells, including STAT3, SRC, AKT, and MAPK signaling [3]. We previously demonstrated that combined targeting of the STAT3 and SRC family pathways significantly increased anti-tumor activity [15]. In this study, we investigated whether this combined targeting could be achieved with FDA-approved drugs. Sunitinib is an FDA-approved tyrosine kinase inhibitor that targets multiple signaling pathways including STAT3 pathway, and dasatinib is a SRC kinase inhibitor [18,21,22,44]. Both of these inhibitors have proven minimally effective in ovarian cancer patients as single-agent therapy, possibly due to compensation by alternative growth/survival pathways. To test the combined effect of these inhibitors on cell signaling pathways in ovarian cancer cells, we treated SKOV3 cells with sunitinib and dasatinib, alone or in combination, for 24 h, then measured the expression of the phosphorylated and total forms of STAT3, SRC, AKT, and MAPK by Western blot (Figure 1B and Figure S1). Treatment with sunitinib alone inhibited activation of STAT3 in SKOV3 cells, which has not been demonstrated previously in ovarian cancer cells. Dasatinib alone inhibited activation of SRC in SKOV3 cells as expected. Combined treatment with both sunitinib and dasatinib led to simultaneous inhibition of p-STAT3 and p-SRC, as well as p-MAPK, which was not inhibited by either alone. However, p-AKT was not inhibited by either single or combined treatment.

### 2.2. Combined Treatment with Sunitinib and Dasatinib Results in a Synergistic Inhibition of Cell Growth/Survival

**Figure 2:**
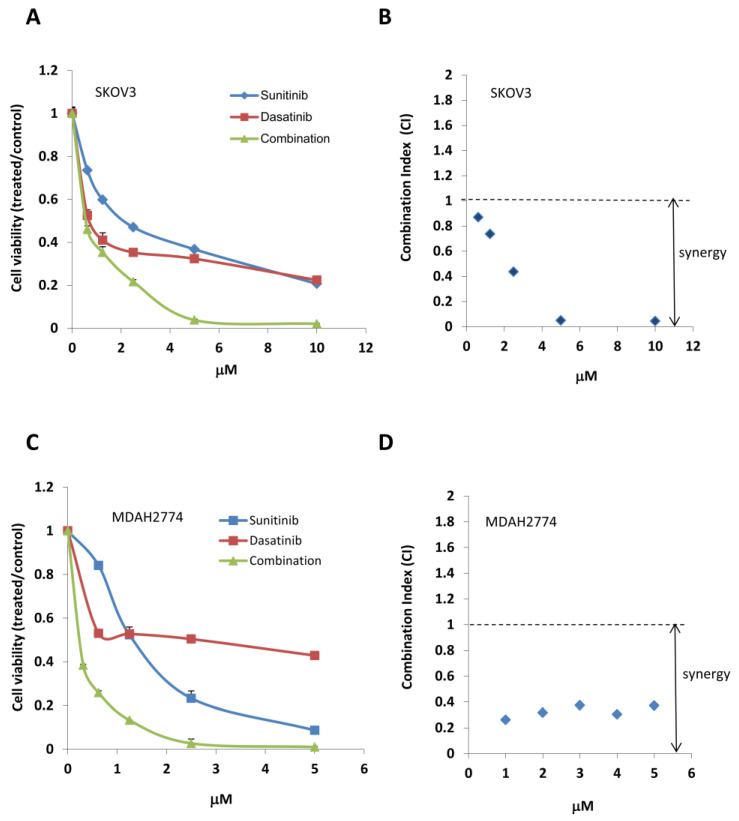
Combined treatment with sunitinib and dasatinib results in synergistic cell growth inhibition. (**A**,**C**) SKOV3 and MDAH2774 cells were treated with sunitinib or dasatinib either alone or in combination at indicated concentrations. Cell viability was determined 72 h later. (**B**,**D**) CI was calculated using the Chou–Talalay method.

We next tested the effect of combined treatment with sunitinib and dasatinib on inhibition of cell viability. We treated SKOV3 cells with sunitinib or dasatinib, either alone or in combination at various concentrations in a fixed molar ratio (1:1). We determined a combination index (CI) using the Chou–Talalay method (CI = 1, additive effect; CI < 1, synergism; CI > 1, antagonism). Combination treatment with sunitinib and dasatinib (SD combination) showed strong synergy, decreasing cell viability much more robustly than either agent alone (Figure 2, Table 1). Strong synergy was also observed in another ovarian cancer cell line MDAH2774. The concentration of sunitinib and dasatinib that gave 75% inhibition (IC75) decreased 5.50-fold and 5.52-fold, respectively, in SKOV3 cells and 4.21-fold and 23.16-fold in MDAH2774 cells (Figure 2, Table 1). We observed a similar synergistic inhibition upon combining sunitinib and dasatinib at other molar ratios (2:1, 5:1, and 10:1; Table 1).

### 2.3. Additional Suppression of the PI3K/AKT/mTOR Pathway Leads to Further Inhibition of Cell Growth

**Figure 3:**
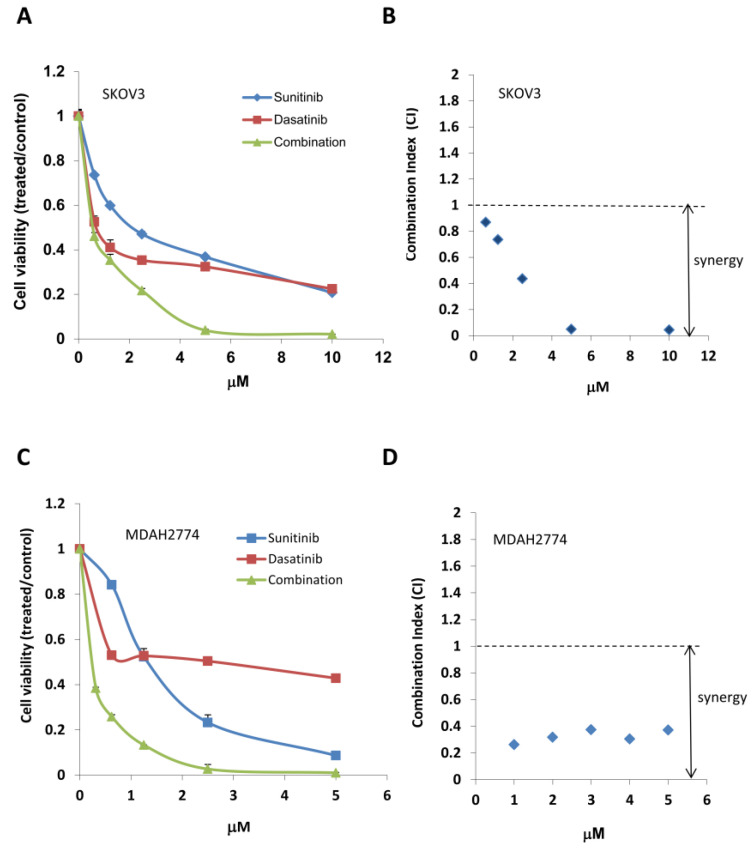
Combined treatment with the AKT inhibitor MK-2206 and both sunitinib and dasatinib results in synergistic cell growth inhibition. (**A**,**C**) MDAH2774 and SKOV3 cells were treated with sunitinib, dasatinib, and MK-2206, either alone or in combination at the indicated concentrations. Cell viability was determined 72 h later. (**B**,**D**) CI was calculated using the Chou–Talalay method.

**Figure 4:**
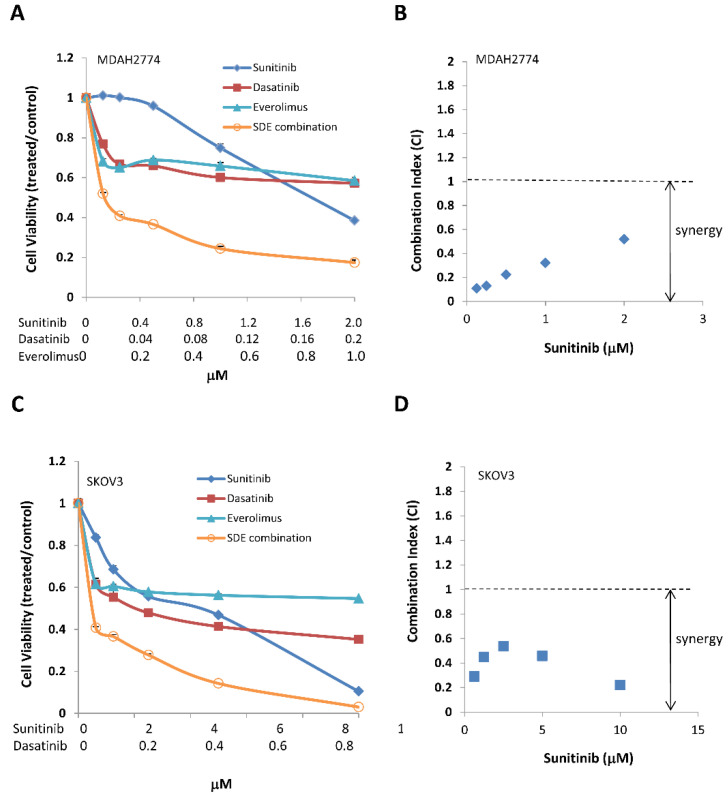
Combined treatment with the mTOR inhibitor everolimus and both sunitinib and dasatinib results in synergistic cell growth inhibition. (**A**,**C**) MDAH2774 and SKOV3 cells were treated with sunitinib, dasatinib, and everolimus, either alone or in combination at the indicated concentrations. Cell viability was determined 72 h later. (**B**,**D**) CI was calculated using the Chou–Talalay method. Results are representative of at least three preparations.

Although combined treatment with sunitinib and dasatinib led to an inhibition of multiple signaling pathways, including STAT3, SRC, and MAPK, the combination had little effect on the PI3K/AKT/mTOR pathway. To determine the effect of additional inhibition of the PI3K/AKT/mTOR pathway on further suppression of tumor growth, we treated MDAH2774 and SKOV3 ovarian cancer cells with a combination of sunitinib and dasatinib, either with or without an AKT inhibitor (MK-2206) or an mTOR inhibitor (everolimus) at various concentrations at a fixed molar ratio, then measured cell viability. Including either an AKT inhibitor (MK-2206) or an mTOR inhibitor (everolimus) led to strong synergy, further decreasing cell viability and the IC50 of each drug in both MDAH2774 and SKOV3 cells (Figure 3 and Figure 4, Table 2 and Table 3). Triple combined treatments were more effective compared to any single treatment or dual combined treatments. Everolimus is an FDA-approved drug, but MK-2206 is currently under clinical investigation. Further study was focused on SDE combination of all FDA-approved drugs (sunitinib, dasatinib, and everolimus). We validated the synergistic inhibition resulting from SDE combination in additional human ovarian cancer cell lines, including A2780CR and OVCAR-8 cells (Table 3). These results demonstrate that simultaneous inhibition of several growth/survival pathways in ovarian cancer leads to better anti-tumor activity compared to inhibiting any of the signaling pathways alone.

### 2.4. Combination Treatment with Sunitinib, Dasatinib, and Everolimus Results in a Synergistic Inhibition of Human Ovarian Tumor Growth in Mice

**Figure 5:**
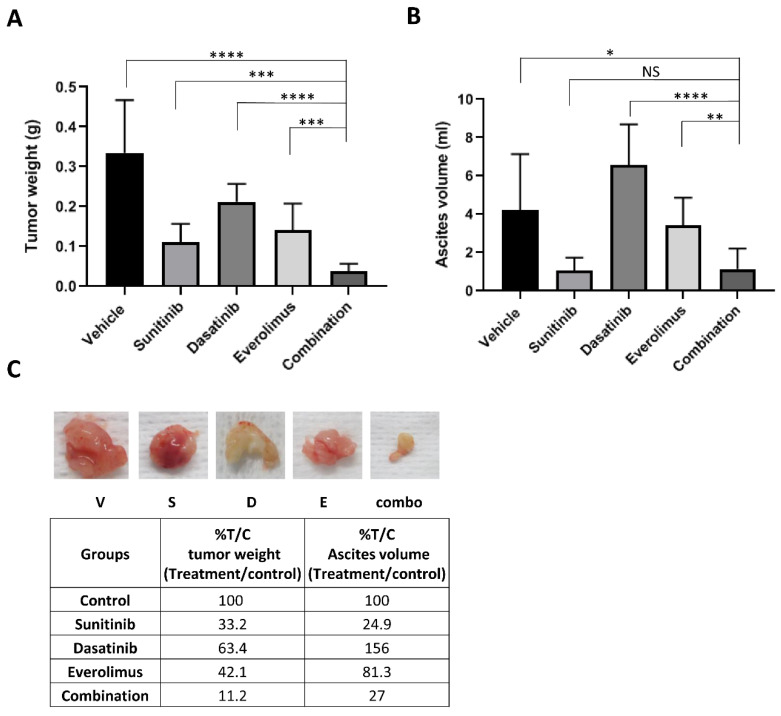
Combined treatment with sunitinib, dasatinib, and everolimus leads to increased inhibition of tumor growth in mice. MDAH2774 cells were implanted into the peritoneal cavities of female athymic nude mice. Tumors were treated with vehicle, sunitinib (40 mg/kg daily), dasatinib (15 mg/kg daily), everolimus (5 mg/kg, 3 times/week) or their combination by oral gavage. Mice were euthanized four weeks later. (**A**) Tumor nodules throughout the peritoneal cavity were excised and weighed. (**B**) Ascites were collected, and the volumes were measured. Data represents mean ± SD (n = 6–10). *, *p* < 0.05; **, *p* < 0.005, ***, *p* < 0.0005, ****, *p* < 0.0001 for combination vs. vehicle, sunitinib, dasatinib, or everolimus alone. (**C**) Representative tumors were photographed, and% tumor weight and ascites volume for each treatment per control (T/C) were calculated.

To assess the ability of the triple combined treatment (SDE) to inhibit tumor growth in vivo more effectively than single treatments, we inoculated athymic nude mice intraperitoneally with MDAH2774 ovarian cancer cells. One week after inoculation, we randomized mice into five groups and treated them with vehicle control, sunitinib, dasatinib, everolimus, or the SDE combination via oral gavage. No toxicity was observed in mice with any of the treatments, as indicated by an absence of significant (>5%) change in body weight Treatment with sunitinib, dasatinib, or everolimus alone decreased average tumor weight from 0.33 g (vehicle control) to 0.11 g, 0.21 g, and 0.14 g, respectively, changed ascites volume from 4.21 mL (vehicle control) to 1.05 mL, 6.57 mL, and 3.45 mL, respectively. The SDE combination treatment further decreased average tumor weight to 0.037 g and reduced ascites volume to 1.14 mL, demonstrating that the combination treatment was more significantly effective than any single treatment (Figure 5). However, further study is needed to address whether this combination can reduce the size of established tumors more effectively.

**Figure 6:**
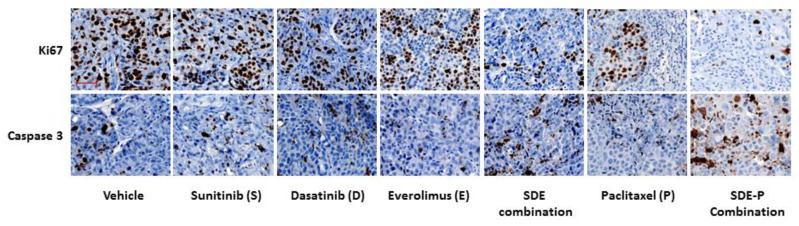
Expression of Ki67 and cleaved caspase-3 by immunohistochemistry (IHC) in tumors. Tumor sections were immunostained with Ki67 and cleaved caspase 3 antibody. Shown are representative images of Ki67 and cleaved-caspase 3 IHC in tumor tissues. Scale bar: 50 μm.

To understand the effect of combined treatment on tumor growth, tumor sections were stained with Ki67 antibody for proliferation and cleaved caspase 3 antibody for apoptosis. As shown in Figure 6, there decreased Ki67 positive cells and increased caspase 3 positive cells in SDE combination group compared to other groups treated with vehicle or each inhibitor alone, indicating that SDE combination inhibited tumor proliferation and induced cell apoptosis in vivo.

### 2.5. SDE Triple Combination Synergistically Increases the Anti-Tumor Activity of Paclitaxel, Both In Vitro and In Vivo

**Figure 7:**
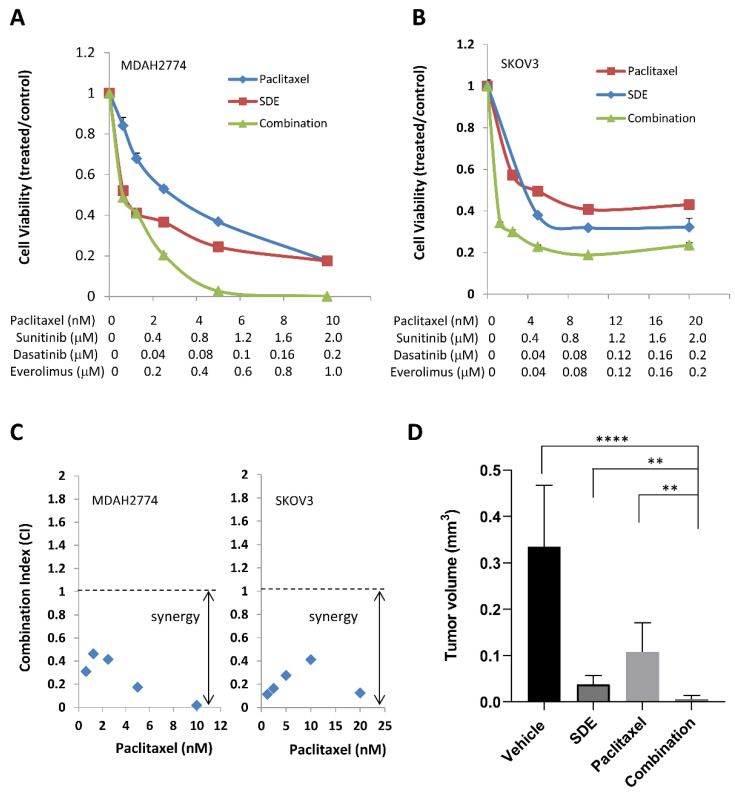
SDE combination increases the anti-tumor activity of paclitaxel, both in culture and in mice. (**A**,**B**) MDAH2774 and SKOV3 cells were treated with paclitaxel together with SDE (sunitinib, dasatinib, and everolimus) combinations, at the indicated concentrations. Cell viability was determined 72 h later. (**C**) CI was calculated using the Chou–Talalay method. Results are representative of at least three preparations. (**D**) MDAH2774 cells were implanted into the peritoneal cavities of female athymic nude mice. Tumors were treated with vehicle, paclitaxel (10 mg/kg, twice/week), SDE combination (sunitinib [40 mg/kg daily], dasatinib [15 mg/kg daily], everolimus [5 mg/kg, 3 times/week]), or combination of all four. Data represents mean ± SD (n = 6–10). **, *p* < 0.005; ****, *p* < 0.0001; combination vs. vehicle, or SDE, or paxlitexel. Mice were euthanized four weeks later. The tumor nodules throughout the peritoneal cavity were excised and weighed.

Although it is a widely used first-line chemotherapy therapy agent for ovarian cancer patients, paclitaxel also causes drug resistance [3,12]. Previous studies suggested that activation of growth signaling pathways confer cell resistance to paclitaxel in ovarian cancer cells [14,45,46,47,48,49]. To determine whether inhibition of growth signaling pathways could enhance the anti-tumor activity of paclitaxel, we incubated human ovarian cancer cells with paclitaxel in the presence or absence of single, dual or triple combinations of sunitinib, dasatinib, and everolimus. We found that any treatment synergistically increased the anti-tumor activity of paclitaxel, with the SDE combination increasing the efficacy of paclitaxel more effectively than any single or dual combination. The IC50 of paclitaxel decreased by 69.5-fold and 6.5-fold in SKOV3 and MDAH2774 cells, respectively, when combined with the SDE combination (Figure 7A–C, Table 4).

Next, we investigated the effect of adding the SDE triple combination to paclitaxel on suppression of tumor growth in mice bearing intraperitoneal MDAH2774 tumors. No toxicity was observed in mice with any of the treatments, as indicated by an absence of significant (>5%) change in body weight Treatment with paclitaxel alone decreased average tumor weight from 0.33 g (vehicle control) to 0.11 g; in contrast, treatment with paclitaxel and the SDE combination further decreased average tumor weight to 0.005 g (Figure 7D), suggesting that the SDE combination could improve the anti-tumor activity of paclitaxel in ovarian cancer.

## 3. Discussion

Preclinical and early clinical studies with single-targeted agents have shown limited antitumor activity in ovarian cancer. Combining two or more therapeutic agents to target tumor cell survival pathways has become a new strategy to treat cancer [16]. In this study, we found that combined treatment of several FDA-approved targeted drugs—sunitinib, dasatinib, and everolimus—results in simultaneous inhibition of multiple signaling pathways and a better anti-tumor activity than any single treatment. In addition, this combination synergistically increases the anti-tumor activity of paclitaxel in ovarian cancer both in vitro and in vivo.

Ovarian cancer is a highly heterogeneous disease. Concurrent activation of multiple signaling pathways, including JAK/STAT3, PI3K/AKT/mTOR, SRC, and MEK/MAPK, appears to be more common in ovarian cancer and might be the critical force that drives ovarian cancer cells to proliferate and survive [12,50]. Treatment of ovarian cancer cells with sunitinib or dasatinib alone blocked phosphorylation/activation of STAT3 and SRC, respectively, but it had little effect on other signaling pathways. Combined treatment with both inhibitors resulted in blockade of both p-STAT3 and p-SRC pathways and additional blockade of p-MAPK, which was not inhibited by either drug alone. The MEK/MAPK pathway is one of the best-characterized signaling cascades and regulates a variety of normal cellular functions, such as cell proliferation, differentiation, and survival [51]. Increased MAPK activity has been reported in ovarian cancer cells. Inhibition of the MEK/MAPK pathway can suppress ovarian cell growth and improve anti-tumor activity of chemotherapy in ovarian cancer cells [52]. Activation of the MEK/MAPK pathway is also observed in ovarian cancer following inhibition of SRC [53]. Dual inhibition of both MEK and SRC has more effective anti-tumor activity in ovarian cancer [54]. Therefore, the synergistic increased anti-tumor activity by combined treatment of sunitinib and dasatinib may be associated with the inhibition of MEK/MAPK signaling pathway. Taken together, our study demonstrated that inhibition of a single pathway might not be sufficient to effectively block ovarian cancer growth and survival when other survival pathways remain active. Combined blockade of multiple growth/survival pathways is more effective against human ovarian cancer than inhibition of any one pathway alone.

Combination of sunitinib and dasatinib led to an inhibition of STAT3, SRC, and MAPK pathways, but had little effect on the PI3K/AKT/mTOR pathway. The PI3K/AKT/mTOR pathway is frequently dysregulated in ovarian cancer and is associated with poor prognosis [55,56]. Although preclinical studies of PI3K/AKT/mTOR pathway inhibitors were promising, the results of early clinical trials were disappointing [57,58,59]. Dual blockade of MEK/MAPK and PI3K/AKT/mTOR pathways has been suggested to be synergistic and more effective [60]. In this study, we demonstrated that additional inhibition of the PI3K/AKT/mTOR pathway led to further suppression of tumor growth. This underscores the concept that simultaneous blockade of multiple cell growth/survival pathways, including JAK/STAT3, PI3K/AKT/mTOR, SRC and MEK/MAPK, may be required to achieve maximum anti-tumor activity in patients with ovarian cancer.

Drug resistance remains one of the major challenges in the treatment of ovarian cancer [12,48,61]. Paclitaxel is a commonly used chemotherapeutic agent in ovarian cancer patients. However, most patients eventually develop resistance to treatment despite high initial response rates. The mechanisms responsible for this acquired chemoresistance are numerous, including the upregulation of multiple cell growth/survival pathways (STAT3, SRC, PI3K, and MAPK) [45,62]. Therefore, combination of targeted agents with paclitaxel is becoming increasing common. In this study, we demonstrated that concurrent inhibition of STAT3, SRC, PI3/AKT/mTOR, and MAPK pathways most effectively improved the anti-tumor activity of paclitaxel both in vitro and in vivo, providing a potential therapeutic strategy to improve clinical benefit of paclitaxel in ovarian cancer patients.

The triple combination of sunitinib, dasatinib and everolimus is most effective against ovarian cancer cell compared to single or dual combination. To our knowledge, this triple combination has not been tested in ovarian cancer either in preclinical or clinical study. Everolimus has been described to improve the efficacy of dasatinib in PDGFRα-driven glioma [63]. The combination of sunitinib and everolimus is currently under phase I study in patients with metastatic renal cell carcinoma. Our results demonstrate that the triple combination represents a novel avenue for the treatment of patients with ovarian cancer. The increased anti-tumor activity by combined treatment could be partially mediated through inhibiting tumor cell proliferation and promoting tumor cell apoptosis as shown in our preliminary results (Figure 6). The increased activity could also be mediated through regulating tumor microenvironment in vivo. More study is needed to understand the mechanisms underlying the synergistic anti-tumor activity by this triple combination.

While the combination of multiple drugs can synergistically increase anti-tumor efficacy, it can also produce unwanted effects. Best combination will be those with lowest effective dose and minimum adverse effect in vivo. Combined treatment can significantly reduce the IC50 of each drug under various molar ratios, thus the dose for each drug in the combination can be reduced. Previous studies with combination of sunitinib and everolimus and combination of dasatinib and everolimus in the patients provide valuable information about the dose for each drug that can be used without causing serious side effect.

## 4. Materials and Methods

### 4.1. Reagents

Sunitinib, dasatinib, MK-2206, AZD-6244, ruxolitinib, and everolimus were purchased from Selleck Chemicals (Houston, TX, USA). Antibodies against p-JAK2 (Y1007/1008), JAK2, p-STAT3 (Y705), STAT3, p-SRC (Y416), SRC, p-AKT (S473), p-MAPK (T202/Y204), MAPK, and GAPDH were obtained from Cell Signaling Technology (Danvers, MA, USA). The antibody against AKT was purchased from Santa Cruz Biotechnology (Dallas, TX, USA).

### 4.2. Cell Culture

SKOV3, A2780CR, and MDAH2774 cell lines were obtained from ATCC (Manassas, VA, USA). OVCAR-8 cells were obtained from the National Cancer Institute (Bethesda, MD, USA). SKOV3, A2780CR and MDAH2774 cells were cultured in DMEM medium and OVCAR-8 cells were cultured in RPMI1640 medium. Culture media were supplemented with 10% FBS and 1% penicillin/streptomycin. All cells were grown in 5% (*v*/*v*) CO_2_ at 37 °C.

### 4.3. Cell Viability Assays

Cells (4000 per well for SKOV3, OVCAR-8, and A2780CR cells; 7000 per well for MDAH2774 cells) were plated in 96-well plate format in 100 µL growth medium for 24 h. Cells were treated with DMSO (vehicle control) or drugs at the indicated concentrations and incubated for an additional 2–3 days. Viable cells were determined using either the MTS assay (Promega, Madison, WI, USA) or the acid phosphatase assay as described previously [64]. For the MTS assay, MTS solution (25 µL) was added directly into each well according to the manufacturer’s instructions. For the acid phosphatase assay, all media was removed; p-nitrophenyl phosphate substrate (10 mM, 100 µL) was added into each well and incubated at 37 °C for 45 min. NaOH was added to stop the reaction, and the absorbance was read at 415 nM. The IC50 was determined using Calcusyn software (Biosoft, Ferguson, MO, USA).

### 4.4. Determination of Combination Index

A combination index (CI) for synergy was determined using the Chou–Talalay method [65] using Calcusyn software. CI values were calculated for the effective doses ED50, ED75, and ED90. A CI value <1 indicates synergy, a CI value >1 indicates antagonism, and a CI value equal to 1 indicates an additive effect.

### 4.5. Western Blot Analysis

Cells were grown in complete medium overnight and treated with DMSO or drugs for 24 h at the indicated concentration. Cells were lysed in RIPA lysis buffer (Thermo Scientific, Waltham, MA, USA) containing Halt protease and phosphatase inhibitors (Thermo Scientific, Waltham, MA, USA). Equal amounts of protein were separated by SDS-polyacrylamide gel electrophoresis. Western blots were performed as described previously [13].

### 4.6. Animal Models

All animal studies were carried out under protocols IACUC11013 approved by the Institutional Animal Care and Use Ethics Committee at City of Hope in accordance with all applicable federal, state, and local requirements and institutional guidelines. MDAH2774 cells (5 × 10^6^) were inoculated into the peritoneal cavity of 6- to 8-week-old female athymic nude mice (National Cancer Institute, Bethesda, MD, USA). Mice were randomized into groups of 10 starting one week after inoculation. Groups were then treated with vehicle (0.5% DMSO in 30% solutol), sunitinib (40 mg/kg oral avage, daily), dasatinib (15 mg/kg, oral gavage, daily), everolimus (5 mg/kg, oral gavage, 3 times a week), a combination of all 3 agents (SDE), paclitaxel (10 mg/kg, intraperitoneal injection, twice a week), or a combination of paclitaxel with SDE. The mice were monitored for ascites production and any adverse effects, then euthanized 3–5 weeks after cell inoculation. Visible tumor nodules were excised and weighed, and the ascites fluid was collected and measured for volume.

### 4.7. Immunohistochemistry (IHC)

Tumor samples were fixed in 10% formalin and embedded in paraffin. IHC was performed using VENTANA Ultra IHC automated stainer (VENTANA Medical Systems, Roche Diagnostics, Indianapolis, IN, USA) as described previously [66]. Slides were scanned with VENTANA iScan HT using VENTANA Image Viewer (VENTANA Medical Systems, Roche Diagnostics, Indianapolis, IN, USA). The images were taken at 20× magnification.

### 4.8. Statistical Analysis

Data are presented as mean ± standard deviation (SD). All experiments were repeated at least three times, each experiment was carried out in triplicate or greater. Student’s *t*-test was used to compare the means of two groups. *p* values less than 0.05 were considered statistically significant.

## 5. Conclusions

Taken together, our results demonstrate that simultaneous blockade of JAK/STAT3, PI3K/AKT/mTOR, SRC and MEK/MAPK pathways results in better anti-tumor activity compared to inhibiting any one or two or three of these signaling pathways. Our study also introduces a novel strategy for inhibiting all these survival pathways in ovarian cancer by combining FDA-approved drugs: sunitinib, dasatinib and everolimus. Finally, our findings provide valuable preclinical information for clinical trials with this potential combination therapy for the treatment of advanced ovarian cancer.

## Figures and Tables

**Table 1:** Synergistic interaction between sunitinib and dasatinib in variety of molar ratios on the viability of ovarian cancer cells.

Ratio Sunitinib: Dasatinib		Fold Reduction (IC50)		Fold Reduction (IC75)		Combination Index (CI)	
		Sunitinib	Dasatinib	Sunitinib	Dasatinib	ED50	ED75
SKOV3	1:1	3.06	0.98	5.50	5.52	1.35	0.36
	2:1	2.52	1.61	4.20	8.41	0.71	0.35
	5:1	2.28	3.63	3.42	17.14	0.59	0.39
	10:1	2.21	7.04	2.82	28.24	1.02	0.36
MDAH2774	1:1	4.96	5.01	4.21	23.16	0.40	0.28
	2:1	4.48	9.03	3.87	42.58	0.34	0.28
	5:1	8.02	15.22	2.67	73.33	0.40	0.39
	10:1	2.96	29.79	2.58	141.94	0.37	0.39

IC50 and IC75: concentrations giving 50% and 75% inhibition; ED50 and 75: effective doses giving 50% and 75% inhibition.

**Table 2:** Synergistic antitumor activity of combining sunitinib (S), dasatinib (D) with MK-2206 (M) in ovarian cancer cells.

Cell	Combination	Fold Reduction (IC50)			Fold Reduction (IC75)			CI	
		S	D	M	S	D	M	ED50	ED75
MDAH2774	SM (1:5)	1.58	NA	7.79	1.05	NA	37.32	0.76	0.97
	DM (1:50)	NA	8.43	37.9	NA	37.44	26.35	0.18	0.06
	SD (10:1)	4.21	9.56	NA	2.19	110.49	NA	0.34	0.46
	SDM (10:1:50)	6.83	15.51	33.68	3.71	186.76	131.44	0.24	0.28
SKOV3	SM (1:1)	5.04	NA	3.76	6.98	NA	26.22	0.46	0.18
	DM (1:10)	4.62	4.40	NA	NA	24.56	17.68	0.44	0.10
	SD (10:1)	3.26	2.3	NA	2.95	15.37	NA	0.74	0.40
	SDM (10:1:10)	7.9	5.6	5.92	10.91	56.84	40.95	0.47	0.13

IC50 and IC75: concentrations giving 50% and 75% inhibition; ED50 and 75: effective doses giving 50% and 75% inhibition; CI: combination index. S: sunitinib; D: dasatinib; M: MK-2206.

**Table 3:** Synergistic antitumor activity of combining sunitinib (S), dasatinib (D) with everolimus (E) in ovarian cancer cells.

Cell	Combination	Fold Reduction (IC50)			Fold Reduction (IC75)			CI	
		S	D	E	S	D	E	ED50	ED75
MDAH 2774	SE (2:1)	6.33	NA	200.1	1.04	NA	529271	0.32	0.097
	DE (1:5)	NA	28.7	825.9	NA	13.01	131663	0.035	0.077
	SD (10:1)	4.21	9.56	NA	2.2	110.42	NA	0.37	0.46
	SDE (10:1:5)	12.8	28.7	814.8	3.2	161.12	1631020	0.11	0.32
SKOV3	SE (10:1)	3.51	NA	29.2	1.58	NA	2811	0.32	0.63
	DE (1:1)	NA	6.2	72.3	NA	3.51	1201	0.18	0.29
	SD (10:1)	3.26	2.3	NA	2.95	15.37	NA	0.74	0.4
	SDE (10:1:1)	6.72	4.76	55.9	4.78	25.15	8591	0.38	0.25
A2780CR	SDE (40:1:2)	26.93	15.06	3.39	5.78	4.59	18.05	0.37	0.44
OVCAR8	SDE (1000:10:1)	5.64	4.95	6.93	2.55	2.53	26.98	0.51	0.82

IC50 and IC75: concentrations giving 50% and 75% inhibition; ED50 and 75: effective doses giving 50% and 75% inhibition; CI: combination index. S: sunitinib; D: dasatinib; E: everolimus.

**Table 4:** Synergistic increased antitumor activity of paclitaxel (P) co-treated with single, dual or triple combinations of sunitinib (S), dasatinib (D), and everolimus (E).

Cells	Combination (Ratio)	Fold Reduction	Combination Index
		IC50 (Paclitaxel)	ED50
MDAH2774	P-S (0.01:2)	1.79	0.72
	P-D (0.01:0.2)	2.27	0.83
	P-E (0.01:1)	2.43	0.41
	P-SD (0.01:2:0.2)	2.4	0.74
	P-SE (0.01:2:1)	3.5	0.46
	P-DE (0.01:0.2:1)	6.3	0.42
	P-SDE (0.01:2:0.2:1)	6.5	0.51
SKOV3	P-S (0.1:10)	3.01	0.39
	P-D (0.1:1)	9.48	0.28
	P-E (0.1:1)	4.15	0.29
	P-SD (0.1:10:1)	6.8	0.85
	P-SE (0.1:10:1)	11.8	0.62
	P-DE (0.1:1:1)	11.1	0.13
	P-SDE (0.1:10:1:1)	69.5	0.02

IC50: concentrations giving 50% inhibition; ED50: effective doses giving 50% inhibition.

## Supplementary Materials

The following are available online at https://www.mdpi.com/2072-6694/12/9/2586/s1, Figure S1: Uncropped Western blot images corresponding to Figure 1B.
