# Supplementary material for: Synergistic Anti-Tumor Activity by Targeting Multiple Signaling Pathways in Ovarian Cancer
Source: Cancers (Basel). 2020 Sep 10;12(9):2586. doi: 10.3390/cancers12092586 (PMC7564386; doi:10.3390/cancers12092586)
Supplement: Supplementary file 1 [file cancers-12-02586-s001.pdf]

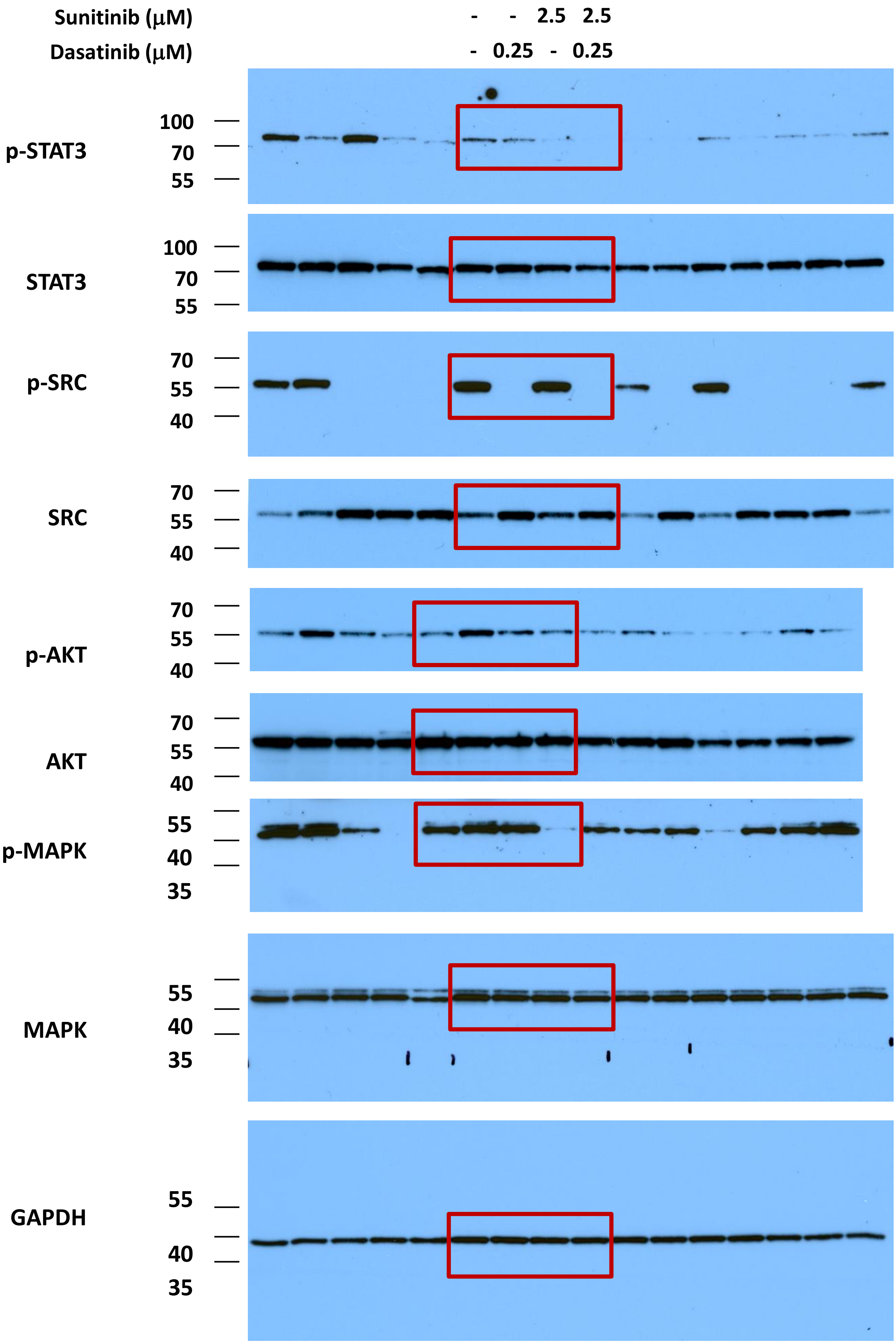

Figure S1. Uncropped Western blot images corresponding to Figure 1B. Red boxes indicate the samples of interest as shown in main text.
